# Risk factors for first-time acute myocardial infarction patients in Trinidad

**DOI:** 10.1186/s12889-018-5080-y

**Published:** 2018-01-19

**Authors:** M. Bahall, T. Seemungal, G. Legall

**Affiliations:** 1 School of Medicine, Faculty of Medical Sciences, University of the West Indies, Eric Williams Medical Sciences Complex, Mt. Hope, Trinidad, Trinidad and Tobago; 2 Trinidad, Trinidad and Tobago

**Keywords:** Risk factors, Trinidad, Acute myocardial infarction, Case–control study

## Abstract

**Background:**

The relative importance of coronary artery disease (CAD) risk varies globally. The aim of this study was to determine CAD risk factors for acute myocardial infarction (AMI) among patients in public health care institutions in Trinidad using a case–control type study design.

**Methods:**

The sample comprised 251 AMI patients hospitalized between March 1, 2011 and April 30, 2012 and 464 age- and sex-matched non-AMI patients with no terminal or life-threatening illness and who did not undergo treatment for CAD. SPSS version 19 was used for data analysis that included chi-square tests, unadjusted and adjusted odds ratios (OR) and conditional multiple binary logistic regression.

**Results:**

There was no difference in age between AMI and non-AMI patients (*p* = 0.551). Chi-square test revealed that clinical and lifestyle variables including stressful life, diabetes, hypertension, hypercholesterolaemia, ischaemic heart disease (IHD), a family history of IHD (*p* ≤ 0.001), smoking (*p* = 0.007) and alcohol consumption (*p* = 0.013) were associated with AMI; sex (*p* = 0.441), ethnicity (*p* = 0.366), age group (*p* = 0.826) and renal failure (*p* = 0.487) were not.

Both unadjusted and adjusted (for age) ORs showed that the odds of hypertension, IHD and alcohol consumption were greater among AMI patients than among non-AMI patients for males; diabetes and IHD for females; and that the odds of a stressful life was greater among non-AMI patients and were the same for both groups with respect to sex, age > 45 years, hypercholesterolemia, renal insufficiency, and family history of IHD.

Conditional multiple logistic regression showed that smoking [OR: 0.274, *p* ≤ 0.001, 95% CI for OR (0.140, 0.537)], a stressful life [OR: 2.697, *p* ≤ 0.001, 95% CI for OR (1.585, 4.587)], diabetes [OR: 0.530, *p* = 0.020, 95% CI for OR (0.310, 0.905)], hypertension [OR: 0.48, *p* = 0.10. 95% CI for OR (0.275, 0.837)] and IHD [OR: 0.111, *p* ≤ 0.001, 95% CI for OR (0.057, 0.218)] were the only useful AMI predictors.

**Conclusions:**

Smoking, diabetes, hypertension, IHD and decrease stress are useful AMI predictors.

## Background

Cardiovascular disease (CVD) is the leading cause of death worldwide [1]. The prevalence of coronary artery disease (CAD), a major contributor to CVD, is related to the increasing prevalence of modifiable risk factors [2]. Previous studies identified diabetes mellitus, hypertension, hypercholesterolaemia, smoking, alcohol consumption, obesity and sedentary lifestyle [3, 4] as risk factors. Other risk factors identified were waist-to-hip ratio, dietary patterns, physical inactivity, blood apolipoproteins, psychosocial factors [5], loneliness and social isolation [6] and C-reactive protein [7], uric acid [8] and homocysteine levels [9]. However, certain risk factors may predominate in certain regions. Smoking is the main determinant of ischaemic heart disease (IHD) amongst the East Indians of Bangalore, India [10] and populations of certain Arab countries [11]. A study conducted in 2016 on Afro-Caribbean persons revealed that the genetic burden for coronary artery disease (CAD) identified on the basis of the 19-single-nucleotide polymorphisms genetic risk score was significantly lower in Afro-Caribbean individuals than in whites [12]. In Trinidad, it has been reported that East Indians are also thought to be at higher risk of CAD by virtue of ‘the migrant gene’ [13, 14]. The present study aimed to compare the association of selected CAD risk factors with acute myocardial infarction (AMI) patients and non-AMI patients using a case–control study design in which patients were matched on the basis of age and sex.

## Methods

This study was a retrospective, observational, case–control study. The case population included all first-time AMI patients (cases) and the control population included non-AMI patients from the same hospital as the cases.

Cases: Patients with a discharge diagnosis of AMI, ST-elevation myocardial infarction, or non-ST-elevation myocardial infarction between 1 March 2011 and 30 April 2012 were identified from the ward registration book. From 2011, AMI patients had additional information documented in an AMI questionnaire used for epidemiological data collection. Case notes of these patients were then reviewed. Confirmed AMI cases, based on the detection of an increase and/or decrease in cardiac biomarkers with evidence of ischaemia with at least one symptom of ischaemia and electrocardiographic changes (new ST-T changes, left bundle branch block or pathological Q waves) [15] were selected for further data extraction. The cardiac biomarker used for confirmation of AMI was Troponin T.

Controls: A convenience sample of non-AMI patients (controls) was selected from among patients admitted in the orthopaedic, surgical, gynaecological and other wards and without a history of treated IHD. Inclusion criteria for non-AMI patients or controls were hospitalization for a problem other than IHD. Exclusion criteria were cases with terminal diseases or major health issues such as end-stage renal disease, human immune deficiency virus infection or cancer. Previous studies [16–18] showed that cases were matched with controls by age and sex. In the present study, AMI patients were matched with either one or two controls by age (± 5 years), as in previous studies [5, 16, 19–21] and sex.

### Setting and sample size

The target population was all adults treated at public hospitals in each of the five regional health authorities (RHAs) in Trinidad and Tobago at the time of the study. The sampled population consisted of adult inpatients at the San Fernando General Hospital during the study period. This hospital accounts for approximately 35% of all hospital admissions in Trinidad and Tobago, and it was selected because ethical approval is granted by this institution.

The required sample size was 524, specifically comprising 262 AMI patients and 262 unmatched non-AMI patients. This sample size was based on an anticipated odds ratio (OR) of 2.0, a 95% confidence level, a relative precision of 50% and an assumption of a 5% probability of exposure to any of the risk factors examined in the study among non-AMI patients. Because the study sought to obtain two matching controls for each AMI patient, the required sample size was found to be 776 or 252 patients and 524 controls [22].

The data collection instrument was an 80-item questionnaire which was administered by trained data collectors (pre-medical students), and face-to-face interviews were conducted for non-AMI patients. The questionnaire comprised questions on type of AMI, timings, patient profile, medical history, social and family history and other clinical information. This is similar to the questionnaire used to extract data from AMI cases. Variables of interest in the study included selected socio-demographics (age, sex, highest level of education and annual income). Lifestyle and clinical variables included self-claimed smoking habit (currently continuing to smoke i.e. up to the year in which interview was conducted) and self-perceived stress (difficulty with coping and anxiousness); family history of IHD (first degree relatives such as siblings and parents usually aged < 50 years [females] or < 55 years [males]) and presence of diabetes, hypertension, hypercholesterolaemia, pre-existing IHD and renal insufficiency.

### Statistical analysis

Microsoft Excel was used to create the database and Version 19 of the Statistical Package for the Social Sciences (IBM SPSS), Version 19.0 for Windows (Armonk, NY: IBM Corp). Both descriptive and inferential data analysis methods were used. Descriptive methods included frequency tables, bar charts for display of age categories and summary statistics (means and standard deviations). Inferential methods included forming 95% confidence intervals for ORs, tests of equality of proportions, chi-square tests of association and conditional logistic regression.

Ethical approval for the study was granted by the Ethics Committee, South West Regional Health Authority and the University of the West Indies.

## Results

### Demographics

**Fig. 1:**
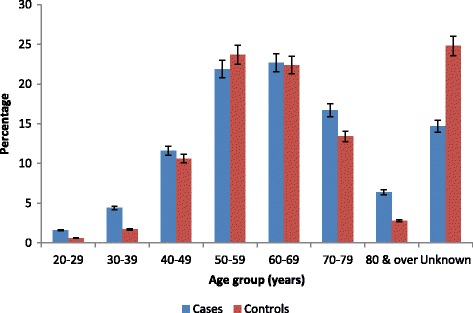
Percentage age group distribution of respondents by patient status IHD, ischaemic heart disease

By the end of the data collection period, 715 of the 786 patients or 91.0% were accepted. Corresponding rates among AMI and non-AMI patients were as follows: 98.7% (251/262) among cases and 88.5% (464/524) among controls. Response rates between AMI and non-AMI patients did not differ significantly among age groups (Fig. 1) or between males and females as well as among ethnic groups. Table 1 shows sex, ethnicity and age group frequency distribution of patients by AMI status. Respondents were primarily males (AMI: 55.0%; non-AMI: 54.1%; overall: 54.4%) and Indo-Trinidadians (AMI: 82.1%; non-AMI: 83.4%; overall: 82.9%) and were aged > 45 years (AMI: 72.5%; non-AMI: 80.6%; overall: 81.2%). The overall mean age of all patients was 59.9 years (standard deviation: 12.07); 60.7 years (standard deviation: 12.66 years) for AMI and 57.4 years (standard deviation: 12.37 years) for non-AMI patients. The difference in the mean age between AMI and non-AMI patients was not statistically significant (*p* = 0.809).

**Table 1:** Frequency and percentage distribution of age and ethnicity of the respondents

	Category: n (%)		
Variable	Cases (AMI)	Control (non-AMI)	*p* value
	(*n* = 251)	(*n* = 464)	
Sex			
Male	138 (55.0)	251 (54.1)	0.875
Female	113 (45.0)	231 (45.9)	0.24
Ethnicity			
Indo-Trinidadian	206 (82.1)	386 (83.4)	0.708
Afro-Trinidadian	45 (17.9)	77 (16.6)	0.654
Age			
20–35	7 (2.8)	15 (3.2)	0.738
36–49	37 (14.7)	75 (16.2)	0.613
50–65	110 (43.8)	211 (45.5)	0.694
65–75	68 (27.1)	121 (26.1)	0.79
> 75	29 (11.6)	42 (9.1)	0.296

The prevalence of selected clinical and lifestyle variables was higher among AMI patients, except for smoking and renal insufficiency for which the prevalence was the same for both groups; in contrast, the prevalence of stressful life was greater among non-AMI patients (Table 2).

**Table 2:** Prevalence of clinical and lifestyle-related risk factors among patients by AMI status

Risk Factor	Cases (AMI)	Control (non-AMI)	*p*-value
	n (%)	n (%)	
Hypertension	185 (74.3)	222 (50.1)	≤ 0.001
Diabetes	158 (63.7)	170 (38.0)	≤ 0.001
IHD	109 (44.1)	27 (8.2)	≤ 0.001
Stressful life	63 (36.6)	231 (56.6)	≤ 0.001
Hypercholesterolemia	49 (19.7)	116 (35.9)	≤ 0.001
FH of IHD	76 (30.3)	13 (2.8)	≤ 0.001
Smoking	85 (34.0)	116 (25.0)	0.050
Alcohol use	81 (32.2)	112 (24.1)	0.019
Renal insufficiency	29 (12.3)	40 (11.9)	0.875

#### Association with cardiovascular risks

Table 3 shows *p* values for tests of the association between ethnicity, sex and age of ≥ 45 years and clinical and lifestyle among AMI and non-AMI patients. Among AMI patients, none of the clinical variables was associated with ethnicity. However, among the non-AMI patients, ethnicity was associated with diabetes (*p* = 0.003) and hypercholesterolaemia (*p* = 0.002). Diabetes (*p* = 0.001), hypertension (*p* ≤ 0.001), IHD (*p* = 0.042), smoking (*p* ≤ 0.001) and alcohol consumption (*p* ≤ 0.001) were associated with the sex of AMI patients; stressful life (*p* = 0.005), hypertension (*p* = 0.005), hypercholesterolaemia (*p* = 0.005), smoking (*p* ≤ 0.001) and alcohol consumption (*p* ≤ 0.001) were associated with the sex of the non-AMI patients. Moreover, age of > 45 years was associated with diabetes (*p* = 0.005), hypertension (*p* ≤ 0.001), smoking (*p* = 0.034) and alcohol consumption (*p* ≤ 0.033) among AMI patients, and with diabetes (*p* ≤ 0.001), hypertension (*p* ≤ 0.001), hypercholesterolaemia (*p* ≤ 0.023) and alcohol consumption (*p* ≤ 025) among non-AMI patient.

**Table 3:** The *p* values for tests of the association with ethnicity, sex and age > 45 years

	Ethnicity		Sex		Age > 45 years	
Variable	Cases	Controls	Cases	Controls	Cases	Controls
Non-modifiable risk factors						
Family history of IHD	0.347	0.380	0.828	0.560	0.512	0.151
Modifiable risk factors						
*Chronic disease*						
Diabetes	0.110	0.003	0.001	0.061	0.010	0.001
Hypertension	0.196	0.163	≤ 0.001	0.005	≤ 0.001	≤ 0.001
Hypercholesterolaemia	0.110	0.002	0.061	0.005	0.940	0.023
IHD	0.776	0.338	0.042	0.622	0.276	0.569
Renal insufficiency	0.875	0.301	0.714	0.210	0.283	0.331
*Lifestyle*						
Smoking	0.077	0.801	≤ 0.001	≤ 0.001	0.016	0.083
Alcohol consumption	0.603	0.105	≤ 0.001	≤ 0.001	0.033	0.025
*Psychosocial stress*						
Stressful life	0.79	0.507	0.085	≤ 0.001	0.062	0.138

Chi-square tests for the association between demographic, clinical and lifestyle variables and AMI revealed that AMI was not associated with sex, ethnicity or renal insufficiency (Table 4).

**Table 4:** Chi-square values, degrees of freedom and *p* values for testing association with AMI

Variable	*χ* ^2^	*df*	*p value*
Demographic variables			
Sex	0.051	1	0.441
Age	1.504	4	0.826
Age > 45 years	3.626	1	0.035
Ethnicity	0.193	1	0.366
Family history of IHD	112.844	1	≤ 0.001
Clinical conditions			
*Chronic disease*			
Diabetes	42.203	1	≤ 0.001
Hypertension	38.489	1	≤ 0.001
Hypercholesterolaemia	19.760	1	≤ 0.001
IHD	101.333	1	≤ 0.001
Renal insufficiency	0.025	1	0.487
*Lifestyle*			
Smoking	6.510	1	0.007
Alcohol consumption	5.467	1	0.013
*Psychosocial stress*			
Stressful life	19.343	1	≤ 0.001

#### Unadjusted and adjusted ORs for age among males and females

Tables 5 and 6 show unadjusted and adjusted ORs, respectively. As seen among all male patients, the odds of hypertension, IHD and alcohol consumption were greater among AMI patients than among non-AMI patients. Among females controlled for age the odds of diabetes mellitus and IHD was higher among AMI cases, and that the odds of reporting to have a stressful life was greater among non-AMI female patients but were the same in both groups for patients aged ≥45 years, regardless of sex, and for patients with at least one of the following diseases or lifestyle habits: hypercholesterolaemia, renal insufficiency, and a family history of IHD.

**Table 5:** Unadjusted and adjusted (for age) odds ratios for male patients

	Odds ratios (Unadjusted)		Odds ratios (Adjusted for age)	
Variable	OR (95% CI for OR)	*p* value	OR (95% CI for OR)	*p* value
Non-modifiable risk factors				
Family history of IHD	2.2 × 10^9^ (0.00, −)	0.997	2.2 × 10^9^(0.00, −)	0.997
Age > 45 years	1.983 (0.635–6.193)	0.239	2.561 (0.593–11.057)	0.208
Modifiable risk factors				
*Chronic disease*				
Diabetes	1.089 (0.496–2.388)	0.832	1.085 (0.494–2.386)	0.829
Hypertension	2.244 (1.011–4.979)	0.047	2.318 (1.033–5.200)	0.041
Hypercholesterolaemia	0.746 (0.289–1.923)	0.544	0.732 (0.278–1.885)	0.508
IHD	7.153 (2.668–19.179)	≤ 0.001	7.275 (2.700–19.601)	≤ 0.001
Renal insufficiency	0.503 (0.154–1.642)	0.255	0.516 (0.158–1.686)	0.273
*Lifestyle*				
Smoking	2.246 (0.986–5.116)	0.054	2.208 (0.970–5.030)	
Alcohol consumption	2.589 (1.176–5.700)	0.018	2.421 (0.398–3.642)	0.035
*Psychosocial stress*				
Stressful life	0.935 (0.432–2.207)	0.866	0.906 (0.415–1.961)	0.805

**Table 6:** Unadjusted and adjusted (for age) odds ratios for female patients

	Unadjusted Odds		Adjusted (for age) Odds	
Variable	OR (95% CI for OR)	*P* value	OR (95% CI for OR)	*p* value
Non-modifiable risk factors				
Family history of IHD	4.7 × 10^9^ (0.000, −)	0.997	24.7 × 10^9^ (0.00, −)	0.997
Age > 45 years	0.040 (0.003, 0.511)	0.013	0.040 (0.00, 0.681)	0.026
Modifiable risk factors				
*Chronic disease*				
Diabetes	4.429 (1.292, 15.182)	0.018	4.436 (1.284, 15.331)	0.019
Hypertension	1.762 (0.491, 6.331)	0.385	1.767 (0.484, 6.454)	0.389
Hypercholesterolaemia	0.383 (0.100, 1.460)	0.16	0.381 (0.096, 1.510)	0.17
IHD	8.103 (1.523, 26.001)	0.002	8.121 (2.137, 30.855)	0.002
Renal insufficiency	1.463 (0.297, 7.211)	0.64	1.463 (0.297, 7.215)	0.64
*Lifestyle*				
Smoking	3.467 (0.385, 31.245)	0.268	3.451 (0.369, 32.279)	0.278
Alcohol consumption	0.714 (0.109, 4.684)	0.725	0.710 (0.104, 4.866)	0.728
*Psychosocial stress*				
Stressful life	0.163 (0.052, 0.516)	0.002	0.163 (0.051, 0.520)	0.002

### Predictors

Finally conditional multiple logistic regression showed that smoking [OR: 0.274, *p* ≤ 0.001, 95% CI for OR (0.140, 0.537)], a stressful life [OR: 2.697, *p* ≤ 0.001, 95% CI for OR (1.585, 4.587)], diabetes [OR: 0.530, *p* = 0.020, 95% CI for OR (0.310, 0.905)], hypertension [OR: 0.48, *p* = 0.10. 95% CI for OR (0.275, 0.837)] and IHD [OR: 0.111, *p* ≤ 0.001, 95% CI for OR (0.057, 0.218)] were the only useful predictors of AMI (Table 7).

**Table 7:** Binary logistic regression (Coefficients and odds ratios)^a^

		OR	*p* value	95% Confidence Interval for OR	
	Regression coefficients			Lower Bound	Upper Bound
Age > 45	− 0.009	0.991	0.434	0.968	1.014
Sex	0.139	1.149	0.655	0.625	2.115
Stressful life	0.992	2.697	≤ 0.001	1.585	4.587
Diabetes	− 0.636	0.530	0.020	0.310	0.905
Hypertension	− 0.734	0.480	0.010	0.275	0.837
Hypercholesterolaemia	0.319	1.375	0.300	0.753	2.513
Ischemic heart disease	−2.196	0.111	≤ 0.001	0.057	0.218
Smoking	−1.294	0.274	≤ 0.001	0.140	0.537
Alcohol consumption	− 0.580	0.560	0.066	0.301	1.040
Model	Likelihood Ratio Tests				
	^b^AIC	−2 log likelihood	chi-Square	df	*P* value
Intercept only	478.539	476.539			
Final	368.224	348.224	128.315	9	≤ 0.001

McFadden *R*^2^ = 0.262

^a^All references values were 0 representing the absence of the condition

^b^Akaike information criterion

## Discussion

Traditional risk factors such as hypertension, diabetes mellitus, history of IHD, family history of IHD, smoking and alcohol consumption, but not stress and hypercholesterolaemia, were associated with AMI. These risk factors were also identified in the Framingham Heart study [3] and the INTERHEART  study [23]. Stress [24] and hypercholesterolaemia [25] have been identified as risk factors in other studies. However, in the present study, these two common risk factors were more prevalent among non-AMI patients. This may have been related to the patients’ unclear understanding and lack of uniformity among patients’ understanding of hypercholesterolaemia confirmation. The lack of an association, though, has also been noted in other studies. Quintana et al. found that hyperlipidaemia was not associated with myocardial infarction-related fatality. [26] In a study conducted by Goldfeld et al., patients with cardiac symptoms without overt CAD showed similar depression and/or stress levels as post-myocardial infarction patients [27]. Hypercholesterolaemia is still a cause of concern since it is related to dietary patterns and type of food consumption [28]. Clusters of risk factors for CAD have been identified in numerous studies in Trinidad. Thomas et al. revealed that diabetes mellitus, hypertension, hyperlipidaemia and cigarette smoking were prevalent among patients presenting with AMI [29]. Mungrue et al. identified smoking and BMI as predictors of AMI-related death or survival [30]. Smoking was associated with a 1.6-times higher risk for AMI and BMI with a 1.3-times higher risk [30]. Alfred et al. found the most common risks associated with AMI in Tobago to be dyslipidaemia, hypertension and diabetes mellitus [31]. None of the local studies were case–control studies. Smoking, diabetes, hypertension and history of CAD were found to be the most common cardiovascular risk factors in Libya [32], and smoking and a family history of CAD were the common ones in Pakistan [33]. In a study on the epidemiology of myocardial infarction, associated risk factors for AMI mortality included age of > 84 years, female sex, educational level and smoking [34].

Subgroup (ethnicity, sex and age) association with cardiovascular risks in our study revealed inconsistent results with AMI and non-AMI cases. However, this study found no difference in the association between ethnicity and AMI status. Ethnic differences in risk factors have been reported by several studies such as those conducted in Singapore and in the UK [35, 36]. Indo-Trinidadians are reportedly at higher risk for CAD than the Indo-British [37]. Ethnic differences in the incidence of AMI may result from factors that were not tested such as high sugar intake, dysfunctional eating, increased waist-to-hip ratio and psychosocial stress although such differences may be less significant owing to the narrowing of lifestyle and cultural differences between the two major ethnic groups in Trinidad and Tobago.

Young patients with AMI demonstrated the additional risk factor of smoking as opposed to their age-matched controls. Among the adults aged < 45 years, the most significant lifestyle-related risk factor was smoking, which is preventable. While age is a non-modifiable risk factor for CAD, there is an increasing young population that suffers from this condition. In this study, the adults aged < 45 years accounted for 8.2% of the patients with AMI, similar to 4 to 10% reported by Harvard Medical School [38]. Those aged < 50 years accounted for 15.3% of the AMI cases. This observation is significant because this signals a possible loss of many years of productive life [39] in addition to the social [40], psychological [41] and physical trauma the patients and their close relatives and dependents experience [42], as well as the economic burden imposed [43].

When the data were analysed according to sex, it was found that diabetes, hypertension, smoking and alcohol consumption were significantly associated with AMI cases. However, stressful life, hypertension, hypercholesterolaemia and smoking showed significant associations among controls. This is in contrast to the findings of Kawano et al. who found that hypercholesterolaemia is an independent risk factor for AMI in men but not in women [44].

Controlling for age, among males, the unadjusted and adjusted OR was higher for hypertension, alcohol and a history of IHD while for females the OR for diabetes mellitus and IHD was higher than non-AMIs. In addition, the odds of reporting a stressful life were greater among non-AMI female patients. A case–control study based on the results of the INTERHEART study showed that hypertension, diabetes, physical activity and moderate alcohol consumption were more strongly associated with myocardial infarction among women than among men [23].

In our study, the predictors for AMI obtained from conditional multiple logistic regression revealed that smoking [OR: 0.274, *p* ≤ 0.001], stressful life [OR: 2.697, *p* ≤ 0.001], diabetes [OR: 0.530, *p* = 0.020], hypertension [OR: 0.48, *p* = 0.10] and IHD [OR: 0.111, *p* ≤ 0.001] were the only useful predictors of AMI. Wilson et al. showed similar predictors diabetes, hypertension, and IHD of AMI [45] in addition to hypercholesterolaemia. In this study, the only modifiable predictable lifestyle factor was smoking. Smoking has become a major public health problem in Trinidad and Tobago. Among the AMI patients aged < 50 years who experienced AMI, 25.5% did not smoke, whereas 31.8% smoked. According to the British-based ERC research firm, over the past 22 years, Trinidad and Tobago has been ranked ninth among the top 10 countries that have recorded the largest percentage increase in cigarette consumption and continues to show an upward trend [46].

### Limitations

The limitations of the study are the small sample size and missing data from patient records. There is an absence of data on dietary intake, including added sugar content, and on psychosocial factors. Information on self-claimed stress and smoking may not meet objective assessment tool or scientific criteria. Patient recall of information can be a problem. Certain risk factors such as obesity could not be obtained because of inadequate information in patient records. Information on hypercholesterolaemia may be questionable because people equate taking lipid-lowering drugs with suffering from hyperlipidaemia or not suffering from the condition if they are not taking lipid lowering therapy.

## Conclusions

Hypertension, diabetes, history of IHD, family history of IHD, smoking and alcohol consumption are associated with CAD. However, the predictors of AMI identified were smoking, diabetes mellitus, hypertension, a history of IHD and decreased stressful lifestyle. There were no significant differences in risk factors between Indo- and Afro-Trinidadians. Effort must be aimed at decreasing lifestyle risks (smoking) and chronic disease risks (diabetes and hypertension) at both the public health and primary care level to curb this epidemic [47]. Early identification of modifiable risk factors is vital to set the strategy for prevention [11]. However, special attention must be paid to smoking, particularly in young individuals.

## Abbreviations

AMI: Acute myocardial infarction
BMI: Basal metabolic index
CAD: Coronary artery disease
CI: Confidence interval
CVD: Cardiovascular disease
IHD: Ischaemic heart disease
OR: Odds ratio
